# mRNA Expressions of Candidate Genes in Gestational Day 16 Conceptus and Corresponding Endometrium in Repeat Breeder Dairy Cows with Suboptimal Uterine Environment Following Transfer of Different Quality Day 7 Embryos

**DOI:** 10.3390/ani11041092

**Published:** 2021-04-11

**Authors:** Ramanathan K. Kasimanickam, Vanmathy R. Kasimanickam

**Affiliations:** 1 Department of Veterinary Clinical Sciences, College of Veterinary Medicine, Washington State University, Pullman, WA 99164, USA; vkasiman@wsu.edu; 2 AARVEE Animal Biotech LLC, Corvallis, OR 97333, USA

**Keywords:** dairy cows, conceptus elongation, embryo quality, endometrial inflammation, mRNA expression, protein

## Abstract

**Simple Summary:**

The mRNA expression of Interferon-τ (*IFNT*), IFN stimulated genes (*ISG15*, *CTSL1*, *RSAD2*, *SLC2A1*, *CXCL10*, and *SLC27A6*), Peroxisome proliferator-activated receptors (*PPARA*, *D*, and *G*), and Retinoid X receptors (*RXRA*, *B*, and *G*) genes and proteins (IFNT, ISG15, CXCL10, PPARG, RXRG, SLC2A1, and SLC27A6) were lower and MUC1 at mRNA and protein levels, was greater in gestation day (GD) 16 embryo and corresponding endometrium of subclinical endometritis cows, and in cows following transfer of poor quality embryo (Grade 3). All genes and proteins but MUC1 expression was lower in GD16 tubular conceptus and corresponding endometrium vs. GD16 filamentous conceptus and matching endometrium in cows with SCE and in cows following the transfer of Grade 3 embryo. Disrupted embryo-uterine communication by altered expression of candidate genes in SCE cows, and in cows following the transfer of poor GD7 embryo negatively programs the conceptus development and plausibly affects the conceptus survival.

**Abstract:**

Effect of the gestational day (GD) 7 embryo quality grade (QG) and subclinical endometritis (SCE) on mRNA and protein expressions of candidate genes [Interferon-τ (*IFNT*), IFN stimulated genes (*ISG15, CTSL1, RSAD2, SLC2A1, CXCL10, and SLC27A6*), Peroxisome proliferator activated receptors (*PPARA, D, and G*), Retinoid X receptors (*RXRA, B,* and *G*), and Mucin-1 (*MUC1*)] in GD16 conceptus and corresponding endometrium were evaluated. After screening of performance records (*n* = 2389) and selection of repeat breeders (*n* = 681), cows with SCE (≥6% polymorphonuclear neutrophils—PMN; *n* = 180) and no-SCE (<6%PMN; *n* = 180) received GD7 embryos of different QGs. Based on GD16 conceptus recovery, cows with SCE (*n* = 30) and No- SCE (*n* = 30) that received GD7 embryos QG1 (good, *n* = 20), 2 (fair, *n* = 20), and 3 (poor, *n* = 20) were included for gene analysis. mRNA and protein expressions (IFNT, ISG15, CXCL10, PPARG, RXRG, SLC2A1, and SLC27A6) differed between SCE and embryo QG groups. All genes but *MUC1* and all proteins but MUC1 expression was greater in filamentous conceptus and corresponding endometrium vs. tubular conceptus and matching endometrium in SCE and embryo QG groups. In conclusion, disrupted embryo-uterine communication by altered expression of candidate genes in SCE cows, and in cows following the transfer of poor embryo negatively programs the conceptus development and plausibly affects conceptus survival.

## 1. Introduction

Proper conceptus development in early gestation is crucial for the successful implantation and maintenance of pregnancy. The morphological transition of the conceptus occurs prior to the implantation in cattle: the conceptus elongates from a sphere to ovoid to tubule to filament [1]. The elongation of the trophoblast provides an increased placental surface area to enable embryo-uterine crosstalk which is essential for the survival and subsequent development of the conceptus. Pregnancy loss is high during initial embryo elongation and this is considered as a crucial developmental period. Although 85% of the breeding results in fertilization, less than 40% of multiparous cows calve after single insemination [2,3,4]. Significant early embryonic loss occurs during the period between fertilization and gestational day (GD) 16 [2,3,4,5,6,7,8]. A substantial proportion of the loss is likely associated with inadequacies in the process of conceptus elongation from GD 9 to 18 [9,10,11]. Although the cause of failure of the embryo to survive and establish pregnancy is multifaceted by paternal, maternal, and embryonic factors [12,13,14], many of the embryonic losses are attributed to maternal factors, such as failure of the uterus to support the conceptus growth and implantation.

The vital changes in the endometrial transcriptome occur between Days 7 and 16 that are primarily regulated by progesterone in diestrus and pregnancy in cattle [15,16,17]. The progesterone concentration in GD 16 is higher in cows that yielded filamentous conceptus compared with the cows that yielded tubular conceptus. These changes in endometrial transcriptome likely establish a favorable uterine environment for the conceptus survival and growth into an elongated, filamentous conformation [15,16,17]. The conceptus elongation is required for the production of Interferon-tau (*IFNT*). The *IFNT* acts on the endometrium to sustain the continued production of progesterone by the ovary and regulates gene expression needed for the conceptus growth [18].

Although much information is known about embryo development, the essential biological pathways important for the conceptus survival and growth remain largely unknown [19]. Understanding the early pregnancy loss has been limited by the lack of defined uterine suboptimal conditions. Several studies have summarized the effect of fertility (high vs. low fertility) on the conceptus growth and survival. Further, we recently used repeat breeder dairy cows with the suboptimal uterine environment (subclinical endometritis) to elucidate the endometrial gene expressions. Candidate genes responsible for embryo elongation were differentially expressed between day 16 endometrium of diestrus and pregnancy, and between subclinical endometritis (SCE) and no-SCE [20,21]. Additionally, the candidate genes involved in the embryo elongation are expressed in greater abundances in GD 16 filamentous conceptus compared to the tubular conceptus (day-matched) in dairy cows [21].

The objective of this study was to investigate the effects of SCE and GD 7 embryo quality on the mRNA expression of candidate genes [*IFNT*, IFN stimulated genes (*ISG15, CTSL1, RSAD2, SLC2A1, CXCL10,* and *SLC27A6*), Peroxisome proliferator activated receptors (*PPARA, D,* and *G*), Retinoid X receptors (*RXRA, B,* and *G*), and Mucin-1 (*MUC1*)] and their proteins expressions in GD 16 conceptus and corresponding endometrium with and without SCE following the transfer of GD 7 embryos with Quality Grades (QG) 1, 2 and 3 in repeat breeder dairy cows. Further, mRNA and protein expressions of candidate genes were explored in filamentous and tubular GD 16 conceptuses relevant to uterine conditions (SCE and no-SCE) and GD 7 embryo quality.

## 2. Materials and Methods

### 2.1. Cows

Upon screening the performance records (*n* = 2389), repeat breeder Holstein cows (*n* = 681) with a history of failing to maintain a pregnancy by three consecutive inseminations and a documented pregnancy loss between 30 and 60 days after any of the first three services post-calving were selected. Endometrial cytology samples [22] from these cows were collected during the luteal phase, two weeks before the embryo transfer, using a Cytobrush Plus GT (Medscand Inc., Hollywood, FL, USA). The cows with subclinical endometritis [SCE, ≥6% polymorphonuclear neutrophils (PMN)] and without SCE (<6%PMN) were included in this study [23]. These cows were in 4th lactation and were at 145 to 190 days postpartum. The selected cows had no history of peripartum metabolic disorders, dystocia, retained placenta, early postpartum uterine diseases, mastitis, or lameness. In addition, these cows were apparently healthy, and their body condition score ranged from 2.5 to 3.5 at initiation of estrus synchronization (5-point score: 1 emaciated to 5 obese) [24,25]. All cows were fed twice daily with a total mixed ration formulated to meet or exceed dietary requirements for lactating Holstein cows weighing ~525 to 740 kg and producing ~26 to 37 kg of 3.5% fat-corrected milk.

### 2.2. Estrus Synchronization

Estrus was synchronized in all cows using a Select-Synch+ CIDR protocol. Briefly, a 1.3 g progesterone intravaginal insert (CIDR, Eazi-Breed™ CIDR^®^ Cattle Insert; Zoetis Animal Health, New York, NY, USA) was placed in the vagina and a 100 µg of gonadorelin diacetate tetrahydrate (GnRH; 2 mL; im; Cystorelin^®^, Merial Inc., Duluth, GA, USA) was administered on Day-10. The CIDRs were removed, estrus alert patches (Western Point Inc., Apple Valley, MN, USA) were fitted, and 25 mg of dinoprost (PGF2α; 5 mL; im; Lutalyse^®^ sterile solution; Zoetis Animal Health) was administered to all cows on Day −3. Cows were observed thrice daily either for standing estrus or for the status of the estrus detector aid up to 96 h. A cow was designated as in estrus if she was visually observed to stand for mounting by her herd-mates or if she had an activated, lost (with mount marks), or partially activated heat detector aid (Day 0).

### 2.3. Embryo Transfer

Cows (SCE, *n* = 180; no-SCE, *n* = 180) that expressed estrus and with an acceptable size of corpus luteum (CL; ≥1.5 cm in diameter) were selected as embryo recipients. All cows were palpated and examined by ultrasonography (7.5 MHz linear transducer, Sonoscape S8, Universal Diagnostic Solutions, Oceanside, CA, USA). Upon administering caudal epidural anesthesia (2.5 to 4.0 mL, 2% lidocaine, MWI Animal Health, Boise, ID, USA), the perineum was cleaned prior to the embryo transfer. Frozen-thawed GD 7 embryos in 1.5 M ethylene glycol were non-surgically transferred into the uterine horn ipsilateral to the CL on Day 7 after estrus. The embryo quality was blocked by the SCE group. The developmental stage of the embryo (4 and 5: Stages 1 to 9; 1, 1-cell embryo to 9, expanding hatched blastocyst), and embryo quality [1, 2 and 3: Codes 1 to 4; 1, excellent/good; 2, fair; 3, poor; 4, unfertilized/dead/degenerate, based on the guidelines of the international embryo transfer society (IETS)] labelled on the straw were recorded at the time of the embryo transfer [26]. 

### 2.4. Embryo Collection

Briefly, all cows (*n* = 360) were flushed by a standard non-surgical uterine flushing technique using an 18-g embryo collection catheter (AgTech Inc., Manhattan, KS, USA) in phosphate buffered saline (PBS; pH 7.4) [27] and GD 16 conceptuses were collected. Conceptuses were then washed in PBS and viewed under the naked eye and a stereomicroscope. The length and width of the embryos were measured. Based on the length, the conceptuses were categorized as tubular (10–20 mm) or filamentous (≥25 mm) [28,29], snap-frozen in liquid nitrogen, and stored at −80 °C.

### 2.5. Endometrial Tissue Sample Collection

Endometrial tissue samples from the ipsilateral uterine horn to the existing CL were collected after the embryo recovery on Day 16 using a Cytobrush Plus GT (Medscand Inc., Hollywood, FL, USA), modified for use in cows [20,21]. One clinician collected all endometrial samples. The samples were snap-frozen and stored at −80 °C until further use. Cows were enrolled in this study as they became available and thus, sample collection occurred on different days/occasions. 

### 2.6. Real-Time Polymerase Chain Reaction for Determination of mRNA Expression of Candidate Genes in Embryo and Endometrial Samples

Candidate gene selection was based on endometrium transcriptome micro-array analysis from the Gene Expression Omnibus (GEO) repository (www.ncbi.nlm.nih.gov/geo, accessed on 10 January 2020) followed by endometrial protein profiling using immunohistochemical data from the Human Protein Atlas portal (www.proteinatlas.org, accessed on 10 January 2020). There, genes were enriched in uterus and embryos which plays a crucial role in embryo elongation signaling.

#### 2.6.1. Total RNA Isolation

Total RNA extraction and complementary DNA synthesis were performed as previously described [20,21]. Approximately 30 mg of embryo and 100 mg of endometrial samples for each class (Figure S1) were used to extract RNA (TRIzol; Invitrogen, Carlsbad, CA, USA). A NanoDrop-1000 spectrophotometer (Thermo Scientific, Rockford, IL, USA) was used to measure the RNA concentration and determine its quality. Sample absorbance was measured after excitation with 260- and 280-ηm light sources. The ratio of absorbance at 260/280 was ~2 (1.99–2.05) for all samples. Before further procedures, all RNA samples were treated with DNAse I (Invitrogen) to remove the DNA contaminant.

#### 2.6.2. Complementary DNA (cDNA) Synthesis

Complementary DNA was synthesized using the iScript cDNA synthesis kit (Bio-Rad Laboratories Inc., Hercules, CA, USA). Approximately 200 to 400 ηg of total RNA based on the sample type was reverse transcribed into cDNA in 20 µL reaction volume. The resulting concentration of cDNA was equivalent to 10 to 20 ηg/µL RNA concentrations. The cDNA was prepared for each pair (embryo and corresponding endometrium) from each biological replicate and stored at −20 °C.

#### 2.6.3. Real-Time PCR of Candidate Genes

Specific primer pairs (Table 1) for gene markers were designed using primer-BLAST (www.ncbi.nlm.nih.gov/tools/primer-blast/, accessed on 10 January 2020). Prior to real-time PCR, ethidium bromide-stained electrophoresis gel for the amplicon of the expected size was performed. Template (cDNA, 2 µL) was amplified for the specific regions of the candidate genes using Taq-PCR master mix in a Hybaid Thermal Cycler, as previously described [20,21]. The DNA template was randomly chosen from samples and a reaction was prepared to amplify the expected fragment of a single gene using a singleplex protocol. Negative controls for the template were also included.

Real-time PCR was done using Fast SYBR Green Master Mix (Applied Biosystems, Foster City, CA, USA), as described [20,21]. Fast SYBR green master-mix (2×) was used to prepare the reaction mix. The final concentration of primers was 0.3 mM in 20 µL of three technical replicates for each sample (2.4 μL of 20 ƞg/μL RNA equivalent cDNA was present in the total volume of three triplicates). A StepOne Plus instrument was used for amplification and melting curve analysis was done for each target marker. Endogenous control bovine ribosomal protein was used to normalize the threshold cycle (CT) values. Fold comparisons were made among tubular and filamentous conceptuses and matching endometrial samples for SCE and embryo QG categories.

### 2.7. Western Blot Analysis

Embryo and endometrium samples obtained from normal and SCE repeat breeder cows were used for Western blot analysis by the method described previously [30]. The embryos and endometrium samples collected on GD 16 were matched for the protein analysis. Briefly, 100 µL of 100% ethanol was added to the organic phase of the sample (from the RNA isolation step). After thorough mixing and incubation, the sample was centrifuged at 2000× *g* at 4 °C for 5 min and the supernatant was further used. 0.5 mL of isopropanol was added to the supernatant and incubated for 10 min. Then the sample was centrifuged at 12,000× *g* at 4 °C for 10 min and the proteins were pelleted. After washing, the protein was resuspended. Further, electrophoresing proteins (60 μg) through 12% SDS-PAGE gel (Bio-Rad Laboratories, Philadelphia, PA, USA), transferring onto polyvinylidene difluoride (PVDF) membrane (Bio-Rad Laboratories), blocking non-specific binding, incubating at 4 °C overnight with primary antibodies (Table S1), washing in wash buffer containing 2% animal serum and 0.1% detergent, and incubating in secondary antibodies (Table S1) conjugated with FITC fluorophore were sequentially performed. Following 1-h incubation with secondary antibodies at room temperature, the membranes were washed. The membranes were scanned using the Pharos FX Plus system (Bio-Rad Laboratories). Conjugated FITC fluorophore was excited at 488 nm and read at the emission wavelength of 530 nm. For each antigen, a single line protein blot was observed from the endometrium and embryo samples. Immunoblots were also probed for glyceraldehyde 3-phosphate dehydrogenase (GAPDH) for the normalization of the samples. Immunoblot lanes were presented in Table S2. After normalization, quantitative protein expression was performed for three separates analyses using the Image J software (National Institutes of Health, Bethesda, MD, USA). The relative protein levels (optical density) were expressed as arbitrary units.

### 2.8. Predicting Functional Protein Partners

Prediction of functional protein partners was analyzed using STRING (https://string-db.org/, accessed on 4 October 2020) to integrate the functions of gene transcripts [31].

### 2.9. Computational Prediction of Protein Targets

Targeted proteins were run through a PANTHER (Protein Analysis Through Evolutionary Relationships) classification system (http://www.pantherdb.org/, accessed on 4 October 2020) to identify the associated biological processes in response to embryo recognition, implantation, and development [32,33].

### 2.10. Statistical Analysis

Data were analyzed using a statistical software program (SAS version 9.4 for Windows, SAS Institute, Cary, NC, USA).

Differences in mean % PMN and days in milk were tested for normality using the Shapiro–Wilk Test. Since the data were found to be normally distributed the mean differences were tested. Differences in mean % PMN and days in milk in repeat breeder dairy cows with or without SCE were determined by ANOVA with Duncan’s multiple range test. 

Real-time PCR and protein data were determined to be normally distributed (Shapiro–Wilk test). The RT-PCR data were analyzed by ANOVA using 2^−ΔΔCt^ values to ascertain the statistical significance of any differences in mRNA abundances of candidate genes among test groups. Initially, differences in relative mRNA abundances in embryo, endometrium, and embryo by endometrium interactions among cows following the transfer of QG 1 (*n* = 20), 2 (*n* = 20), and 3 (*n* = 20) embryos were determined. Further, differences in relative mRNA abundances in embryo, endometrium, and embryo by endometrium interactions in cows with (*n* = 30) versus without (*n* = 30) subclinical endometritis were determined. In addition, differences in relative mRNA abundances in the embryo (tubular and filamentous) and corresponding endometrium following the transfer of QG 1, 2, and 3 embryos in cows with versus without subclinical endometritis were elucidated. Similarly, to determine differences in protein levels among test groups, mean values were analyzed by the general linear model, and pair-wise comparisons were done with a Tukey’s honest significant difference test. 

It was hypothesized that two-fold mean differences in mRNA expressions are biologically relevant. To detect these differences with adequate statistical power (1 − β = 0.8) and statistical significance (α = 0.05), at least five samples per group were needed.

## 3. Results

Mean (±SEM) % PMN differed (*p* < 0.05) between cows with or without SCE, 2.8 ± 1.0 and 11.2 ± 3.4, respectively. Mean (±SEM) days in milk did not differ between cows with or without SCE, 162.4 ± 18.2 and 155.9 ± 13.2, respectively (*p* > 0.05).

Out of 360 cows that received GD7 embryos, 152 (42%) embryos were recovered on GD16. Of those recovered, intact conceptus recovery was 91.9% for conceptuses measured up to 40 mm in length, 85.6% for conceptuses measured from 41 mm to 60 mm, and 69.1% for conceptuses measured above 60 mm. Completely fragmented conceptuses beyond the level of measurement of length were 4.3%. Total percentage of GD16 conceptuses recovered differed between cows with SCE and with no-SCE, 36.1% (65/180) and 48.9% (87/180), respectively (*p* < 0.05). Total percentage of GD16 conceptuses recovered following transfer of good (53.3%; 64/120; *p* < 0.001) and fair (44.2%; 53/120; *p* < 0.01) quality GD7 embryos were higher compared with transfer of poor quality GD7 embryos (29.2%; 35/120). The percentage of GD16 embryo recovered were similar following transfer of good and fair GD 7 embryo (*p* > 0.1). The total number of filamentous conceptuses recovered was lower for SCE cows compared with no-SCE cows (*p* < 0.01; 15.0% vs. 25.6%). Total number of tubular conceptuses recovered did not differ between SCE and no-SCE cows (*p* > 0.1; 21.1% vs. 22.8%). Total percentage of GD16 filamentous conceptuses recovered following transfer of poor quality embryo was lower compared to good quality embryo (*p* < 0.01; 23.3% vs. 55.0%) and fair quality embryo (*p* < 0.05; 23.3% vs. 43.3%). Percentage of GD16 filamentous embryo recovered following transfer of Stage 4 and 5 embryos did not differ, 46.6% (34/73) and 53.4% (39/73), respectively (*p* > 0.1). Percentage of GD16 tubular embryo recovered following transfer of Stage 4 and 5 embryos did not differ, 53.2% (42/79) and 46.8% (39/73), respectively (*p* > 0.1).

### 3.1. mRNA Abundances in GD 16 Conceptus and Endometrium of Cows that Received Embryo with Quality Grade 1, 2 and 3

**Figure 1:**
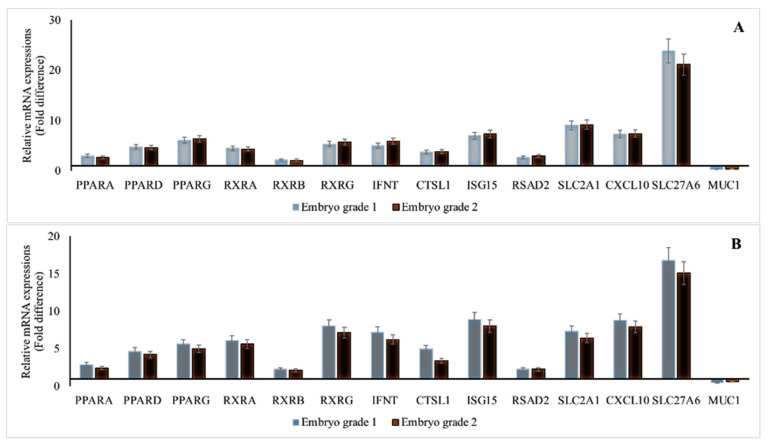
mRNA abundances in gestational day (GD) 16 embryo (**A**) and corresponding endometrium (**B**) of repeat breeder cows ^1^ following transfer of GD 7 embryo with Quality Grades (QG) ^2^ 1, 2 and 3 (*). * Reference group (embryo QG 3) relative mRNA expression is 1-fold; all candidate genes were upregulated and MUC 1 was down regulated compared to reference group **(***p* < 0.05); ^1^ repeat breeder cows were with the history of failing to conceive after the first three inseminations post calving and documented pregnancy loss between 30 and 60 days following any of the first three services. ^2^ Embryo QG: Codes 1 to 4; 1, excellent/good; 2, fair; 3, poor; 4, unfertilized/dead/degenerate); *PPAR*—peroxisome proliferator-activated receptor; *RXR*—retinoid X receptor; *IFNT*—interferon-τ; *CTSL1*—Cathepsin L 1; *ISG15*—interferon-stimulated gene-15; *RSAD2*—Radical S-Adenosyl Methionine Domain Containing 2; *SLC2A1*—Solute Carrier Family 2 Member 1; *CXCL10*—C-X-C Motif Chemokine Ligand 10; *SLC27A6*—SoluteCarrier Family 27 Member 6; *MUC1*—Mucin 1.

The mRNA abundances of the genes except *MUC1* were greater whereas the gene abundance of *MUC1* was lower in GD 16 conceptus (Figure 1A) and corresponding endometrium (Figure 1B) of cows that received GD 7 embryo QG 1 and 2 compared with cows that received GD 7 embryo with QG 3 (*p* ≤ 0.05).

### 3.2. mRNA Abundances in GD 16 Conceptus and Endometrium of Cows with or without Subclinical Endometritis

**Figure 2:**
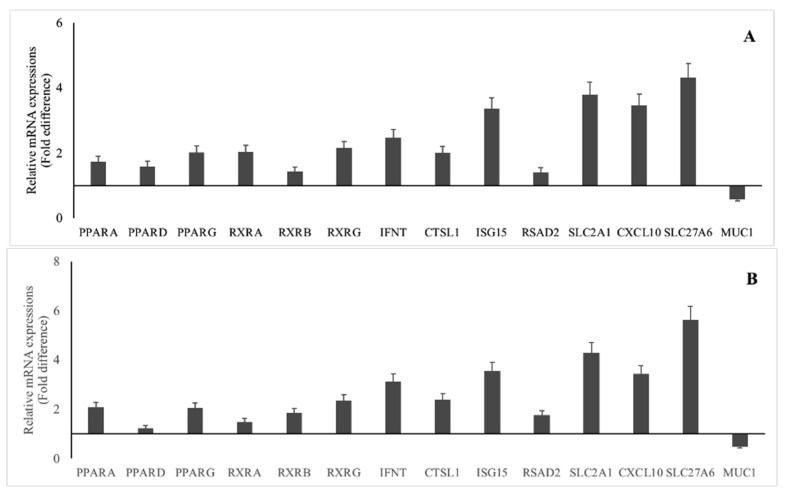
mRNA abundances in gestational day (GD) 16 embryo (**A**) and corresponding endometrium (**B**) of repeat breeder cows ^1^ with (*) or without subclinical endometritis ^2^. * Reference group, relative mRNA expression is equal to one fold; all candidate genes were upregulated and MUC 1 was down regulated compared to reference group (*p* < 0.05); ^1^ repeat breeder cows were with the history of failing to conceive after the first three inseminations post calving and with the history of at least one pregnancy loss between 30 and 60 days after any of the first three services during the second or third lactation; ^2^ cows with SCE, >6% PMN; cows without SCE, ≤6% PMN; *PPAR*—peroxisome proliferator-activated receptor; *RXR*—retinoid X receptor; *IFNT*—interferon-τ; *CTSL1*—Cathepsin L 1; *ISG15*—interferon-stimulated gene-15; *RSAD2*—radical S-adenosyl methionine domain containing 2; *SLC2A1*—Solute Carrier Family 2 Member 1; *CXCL10*—C-X-C Motif Chemokine Ligand 10; *SLC27A6*—Solute Carrier Family 27 Member 6; *MUC1*—Mucin 1.

The mRNA abundances of the genes were greater whereas the gene abundance of *MUC1* was lower in GD 16 conceptus (Figure 2A) and corresponding endometrium (Figure 2B) of cows without SCE compared with cows with SCE (*p* ≤ 0.05).

### 3.3. Interactions between Subclinical Endometritis Status by Embryo Quality Grade mRNA Expressions

Interaction between the subclinical endometritis status by embryo QG for mRNA expressions was observed. *p*-values of pairwise comparisons of mRNA expressions in GD 16 conceptus and corresponding endometrium in repeat breeder cows following the transfer of different GD 7 embryo QG are given in Table 2a–d.

### 3.4. mRNA Expressions of GD 16 Filamentous vs. Tubular Conceptus of Cows with or without Subclinical Endometritis Following Transfer of Different GD 7 Embryo Grades

**Figure 3:**
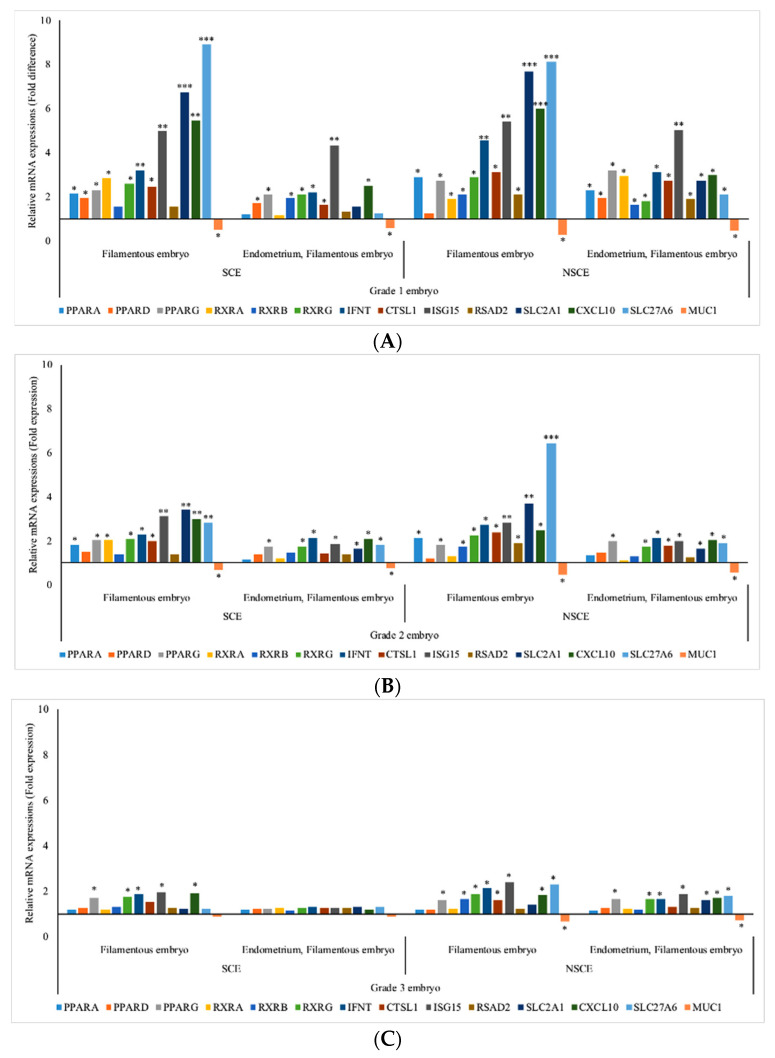
mRNA abundances (mean ± SEM) in gestational day (GD) 16 filamentous ^1^ conceptuses (compared to tubular conceptuses ^§^) and corresponding endometrium (compared to endometrium that yielded tubular ^1^ conceptuses ^§^) from repeat breeder cows ^2^ with or without subclinical endometritis ^3^ following transfer of GD 7 embryo Quality Grade ^4^ (QG) 1 (**A**), embryo Grade 2 (**B**) and embryo Grade 3 (**C**). * *p* < 0.05; ** *p* < 0.01; *** *p* < 0.001; ^§^ Reference group, relative mRNA expression is equal to 1 fold; ^1^ Filamentous conceptus, ≥25 mm long; Tubular conceptus, 10 to 20 mm long; ^2^ Repeat breeder cows were with the history of failing to conceive after the first three inseminations post calving and with the history of at least one pregnancy loss between 30 and 60 days after any of the first three services during the second or third lactation; ^3^ Cows with subclinical endometritis, >6% PMN on endometrial cytology; cows without subclinical endometritis, ≤6% PMN on endometrial cytology; ^4^ Embryo QG: Codes 1 to 4; 1, excellent/good; 2, fair; 3, poor; 4, unfertilized/dead/degenerate); *PPAR*—peroxisome proliferator-activated receptor; *RXR*—retinoid X receptor; *IFNT*—interferon-τ; *CTSL1*—Cathepsin L 1; *ISG15*—interferon-stimulated gene-15; *RSAD2*—Radical S-Adenosyl Methionine Domain Containing 2; *SLC2A1*—Solute Carrier Family 2 Member 1; *CXCL10*—C-X-C Motif Chemokine Ligand 10; *SLC27A6*—Solute Carrier Family 27 Member 6; *MUC1*—Mucin 1.

The mRNA abundances were differentially expressed in the filamentous conceptus and corresponding normal, and subclinical endometrium compared with the tubular conceptus and corresponding normal, and subclinical endometrium following the transfer of different grades of GD 7 embryos (Figure 3).

### 3.5. Protein Expression in GD 16 Conceptus and Corresponding Endometrium

**Figure 4:**
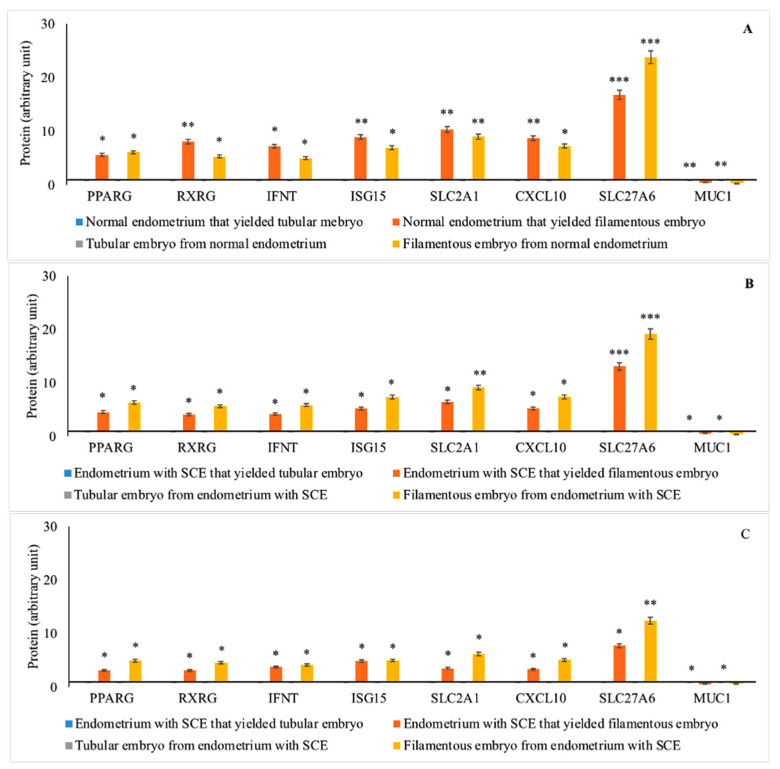
Protein abundances (mean ± SEM) in gestational day (GD) 16 filamentous conceptus^1^ and corresponding endometrium (compared to tubular conceptus ^1^ and corresponding endometrium §) from repeat breeder ^2^ cows with or without subclinical endometritis ^3^ following transfer of GD 7 embryo Quality Grade ^4^ (QG) 1 (**A**), embryo Grade 2 (**B**) and embryo Grade 3 (**C**). § Reference group, relative mRNA expression is equal to 1 fold; * *p* < 0.05; ** *p* < 0.01; *** *p* < 0.001; ^1^ Filamentous conceptus, ≥25 mm long; Tubular conceptus, 10 to 20 mm long; ^2^ repeat breeder cows were with the history of failing to conceive after the first three inseminations post calving and with the history of at least one pregnancy loss between 30 and 60 days after any of the first three services during the second or third lactation; ^3^ Cows without subclinical endometritis, ≤6% PMN on endometrial cytology; ^4^ Embryo QG: Codes 1 to 4; 1, excellent/good; 2, fair; 3, poor; 4, unfertilized/dead/degenerate; PPAR—peroxisome proliferator-activated receptor; RXR—retinoid X receptors; IFNT—interferon-τ; ISG15—interferon-stimulated gene-15; SLC2A1—Solute Carrier Family 2 Member 1; SLC27A6—Solute Carrier Family 27 Member 6; CXCL10—C-X-C Motif Chemokine Ligand 10; MUC1—Mucin 1.

The mean (± SEM) relative protein levels (optical density) were stated as arbitrary units. The expressions of IFNT, ISG15, CXCL10, PPARG, RXRG, SLC2A1, and SLC27A6 proteins were greater and MUC1 protein was lower for embryo QG 1 and 2 compared with embryo QG 3. Similarly, expressions of IFNT, ISG15, CXCL10, PPARG, RXRG, SLC2A1, and SLC27A6 proteins were greater and MUC1 was lower in the endometrium of cows without endometritis compared with cows with endometritis (*p* < 0.05). Interaction between subclinical endometritis status by the embryo QG for protein expressions was observed. *p*-values of pairwise comparisons of protein expressions in GD 16 conceptus and corresponding endometrium following the transfer of different GD 7 embryo QG were given in Table 3. Protein abundances were different among filamentous conceptuses and corresponding normal endometrium compared with tubular conceptuses and corresponding subclinical endometrium following the transfer of GD 7 embryos of different QG (Figure 4).

### 3.6. Interactive Pathway of Genes Associated with Embryo-Uterus Crosstalk on GD 16

**Figure 5:**
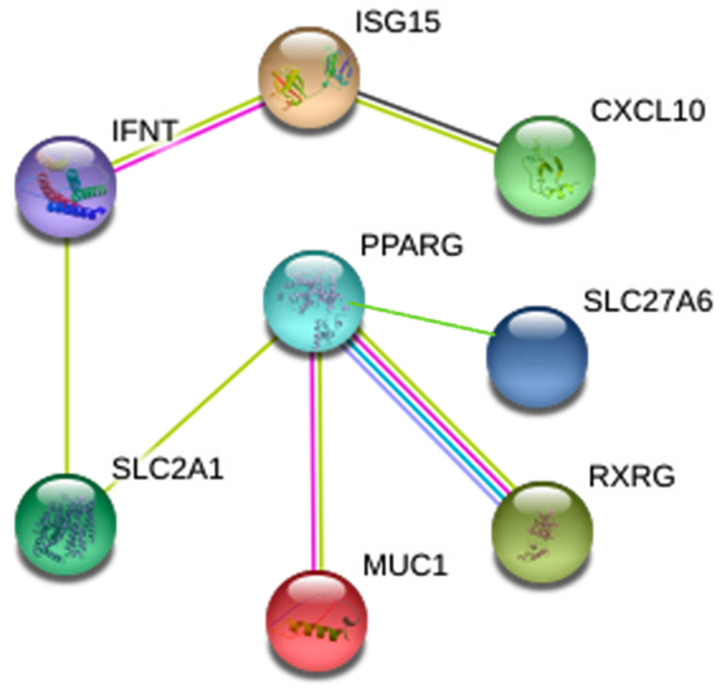
Simple interactive pathway of genes on gestational Day 16 of bovine pregnancy.

All functional proteins investigated in this study were submitted for the analysis of their pathways and interactions (Figure S2). There were interactions of IFNT, ISG15, CXCL10, PPARG, RXRG, SLC2A1, SLC26A7 and MUC1, presented as a simple interactive pathway (Figure 5).

### 3.7. Computational Prediction of Protein Targets

**Figure 6:**
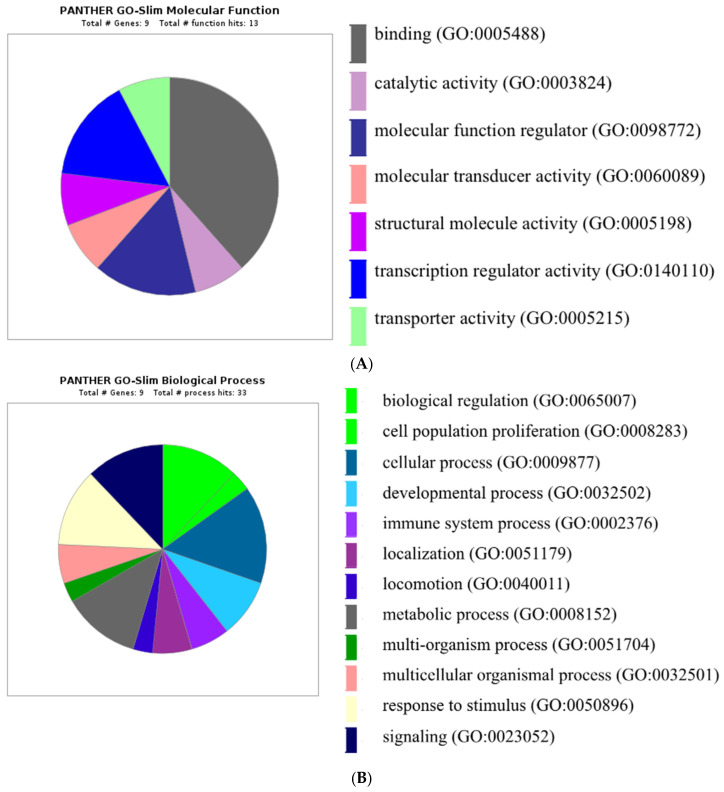

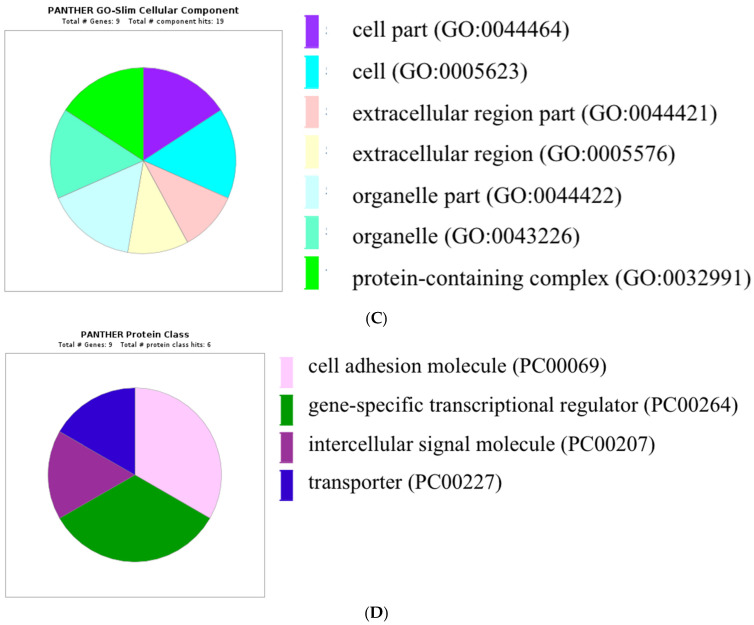
Gene ontology (GO) processes of target proteins depicting (**A**) Molecular functions; (**B**) Biological process; (**C**) Cellular component and (**D**) Protein class

Gene ontology of key protein targets accountable for embryo development and elongation are shown in Figure 6. The differentially expressed key protein had 13 predicted molecular function hits, involving binding, catalytic activity, molecular regulator function and activity, structural molecule activity, transporter regulated activity and transporter activity; the differentially expressed key proteins had 33 biological processes hits, comprising of biological regulation, cell proliferation, cellular process, developmental process, immune system process, localization, locomotion, metabolic process, multi-organismal process, multi-cellular organismal process, response to stimulus and signaling. The differentially expressed key proteins had 19 cellular components hits, consisting of cell, extracellular region, organelle, cell part, extracellular region part, organelle part, and protein-containing complex. The differentially expressed key proteins had six predicted protein class hits, involving cell adhesion molecule, gene-specific transcriptional regulator, intercellular signal molecule and transporter.

## 4. Discussion

In the current study, mRNA expressions of all candidate genes evaluated were greater in abundances, but *MUC1* gene abundance was lower in both in GD 16 filamentous conceptus and corresponding endometrium in cows without SCE than in tubular conceptus and corresponding endometrium in cows with SCE. In our previous study, following artificial insemination, we observed mRNA abundances of candidate genes were greater in the filamentous conceptus and corresponding endometrium in cows without SCE than in the tubular conceptus and corresponding endometrium in cows with SCE on GD 16, with the exception that *RSAD2*, *PPARA*, and *RXRA* did not differ between the filamentous conceptus and corresponding endometrium in cows without SCE and the tubular conceptus and corresponding endometrium in cows with SCE [21]. Further, in addition to IFNT, ISG15, CXCL10, PPARG, RXRG, protein expressions of SLC2A1 and SLC26A7 were greater in the filamentous conceptus and the corresponding endometrium without SCE compared to the tubular conceptus and corresponding endometrium with SCE following the transfer of GD 7 embryo in this present study. However, SLC2A1 and SLC26A7 proteins expression did not differ between GD 16 filamentous conceptus and corresponding endometrium without SCE and GD 16 tubular conceptus and corresponding endometrium with SCE in repeat breeder cows following artificial insemination (AI) [21]. The optimum uterine environment is one of the crucial and important elements for the successful establishment of pregnancy in cows. Genes are seven times upregulated or downregulated in a receptive endometrium compared to a nonreceptive endometrium between Days 7 and 14 of the estrous cycle [34]. Altering the embryo culture environment with the products of endometrial inflammation had a profound effect on embryo quality by hindering trophoblast cell proliferation [35]. It should be noted that with an increase in inner cell mass, trophoblast ratio may have plausibly affected embryonic survival.

Despite the strong evidence of the role of candidate genes on embryo elongation, embryos developed from different applied reproductive technologies [AI, in vivo embryo production, in vitro embryo production (IVP), and somatic cell nuclear transfer (SCNT)] showed differences in these candidate transcriptomes. These differences in gene expression consequentially influence embryo elongation. Gene expression analysis of GD 16 bovine embryos showed that 365 genes were differentially expressed in IVP embryos compared to embryos produced from AI [36]. Differentially expressed transcripts in GD 16 conceptuses produced in vitro versus from AI were highly correlated to the functional networks of glucose and lipid metabolism, cellular movements, and cellular growth and proliferation [36]. It is plausible that the embryo quality also incriminates the observed differences in the conceptus length and its functional genes. The mRNA abundances of the candidate genes were greater in the GD 16 conceptus for QG 1 and 2 GD 7 embryos compared to the QG 3 GD 7 embryo in the current study. Studies that investigated the blastocyst size (based on cell number) and subsequent embryo development after hatching, revealed that conceptuses derived from the transfer of large blastocysts were longer than those derived from small blastocysts [37]. It should be noted that the embryo QG is based on morphological integrity of embryos, 85, 50, and 25% cellular integrity for QG 1, 2, and 3, respectively [26].

Embryo-maternal interaction may occur as early as GD 8 of pregnancy in cattle [38]. Exposure of endometrial explants to GD 8 blastocyst in vitro for 6 or 24 h induced the expression of *ISG*s including *RSAD2* [39]. Endometrial expression of *RSAD2* was influenced by IFNT secretion from the conceptus during early pregnancy in cattle [40,41]. RSAD2 protein contains a radical S-adenosylmethionine (SAM) domain that catalyzes diverse reactions required for embryo development. Radical SAM proteins are utilized in DNA precursor, vitamin, cofactor, antibiotic and herbicide biosynthesis, and biodegradation pathways [42]. These proteins synthesized in endometrial cells during the peri-implantation period could play a pivotal role to support the conceptus development. *RSAD2* along with *IFIH1* is implicated in establishing RNA interference, an antiviral mechanism that can modulate innate immune responses and potentially contribute to maternal recognition, embryo elongation, and implantation [43]. Along with other genes, *PPARA* and *RXRA* are implicated in maintaining cellular morphology, cellular arrangement, and cellular movements of developing embryos orchestrated by mechanochemical feedback [44,45]. This feedback coordinates cross-talks between cells and instructs cells to self-organize by cell fate decision and patterning and architecture of developing embryo [44,45]. Interestingly, differentially expressed transcripts in elongated embryos generated by IVP vs. AI, included *PPARA* and *RXRA* [36]. The differential expression of mRNAs of *RSAD2*, *PPARA,* and *RXRA* following AI or transfer of Day 7 embryo as noted in our studies indicated that these candidate genes could potentially play a supportive role in embryo development when the embryos are derived from different applied reproductive techniques. 

The microenvironment of the uterine lumen is critical for the survival and development of the peri-implantation conceptus [46]. In the uterine lumen, the amount of recoverable glucose substantially increases between the GD 10 and 16. Glucose is one of the major sources of energy for developing conceptus [47], and it is utilized for hypertrophy, hyperplasia, and migration of trophectoderm cells [48]. The *IFNT* triggers the expression of several progesterone-induced genes that encode transporters of glucose including *SLC2A1* in the endometrium of ruminants [49]. 

In addition, the process of conceptus elongation requires a high demand for lipids [50]. Lipids are available in the uterine fluid to be used by conceptus cells [50,51,52]. The presence of an elongating conceptus promotes changes in the uterine lipid profile that may be due to lipid metabolism transduction events occurring during elongation [50,51,52]. Fatty acids provide energy to support the requirements of the proliferating tissue [53]. Trophectoderm cells during elongation, are very active and secrete considerable amounts of bioactive factors, which also require energy to be synthesized [21]. During the conceptus elongation, increased *SLC27A6* expression is highly correlated with the expression of *PPARG* which is a critical gene for the uptake, activation, synthesis, and modification of fatty acids [47,48]. Largely, these findings suggest the important role of lipids in the process of elongation of the preimplantation conceptus in ruminants. Dietary supplementation with fat in general and specifically with ω3 fatty acid has been shown to improve fertility in bovines [54]. Pregnant repeat breeder dairy cows supplemented with fish oil rich in eicosapentaenoic acid and docosahexaenoic acid from −2 to +2 weeks of artificial insemination showed a greater abundance of *ISG15*, *RTP4*, *Mx2,* and *OAS1* transcripts in peripheral leukocytes [55]. Angus heifers were supplemented with either 450 g of rumen-protected fish oil (omega 3 FA) or sunflower oil (omega 6 FA) for 8 weeks, starting 5 weeks prior to breeding synchronization. Interestingly, the embryo elongation was enhanced in the omega 3 group compared to the omega 6 group [56]. 

The differential expression of genes in the current study potentially explains the effect of embryo quality and uterine environment on embryo elongation (filamentous vs. tubular) and survival. Further, the results from the current and previous studies explain how differences in gene expression impact embryo development when the embryo is produced in different environments (AI vs. in vivo embryo). The study that compared elongated embryos from SCNT and IVP, and artificial insemination used only morphologically sound IVP blastocyst for embryo transfer [36]. However, in the current study, in vivo embryos of three different QG were used to transfer into the recipient cows. Interestingly the genes responsible for embryo elongation were differentially expressed in GD 16 conceptus, with greater abundances in embryo QG 1 and 2 compared with embryo QG 3. 

We observed that the % conceptus recovered on GD 16 differed between cows with and without SCE, 36.1 vs. 48.3% respectively (*p* < 0.05) [57]. Similarly, the percentage conceptus recovered on GD 16 differed following the transfer of GD 7 embryo QG 1, 2, and 3, 53.3, 44.2, and 29.2%, correspondingly (*p* < 0.001). The % GD 16 conceptus recovery was similar following the transfer of QG 1 and 2, but the % GD 16 conceptus recovery following the transfer of QG 1 (*p* < 0.001) and 2 (*p* < 0.01) GD 7 was greater compared with the transfer of the QG 3 GD 7 embryo. Further, GD 7 embryo QG and uterine inflammation significantly influenced the length, width, and weight. Interestingly, progesterone concentration on GD 16 was greater in cows with filamentous conceptus compared to the cows with tubular conceptus [57,58]. It is evident that the SCE and in vivo embryo QG influenced the embryo development between GD 7 and GD 16 by altering the cross talk between the uterus and embryo by modifying the expression of candidate genes and specific proteins. 

Gene ontology analysis for the upregulated genes in embryo and/or endometrium revealed various functional terms indicating that these genes are associated with the embryo survival, implantation, and development. The associated functions of the protein candidates were involved with the process of conceptus elongation [59,60,61]. Differential response from the endometrium revealed extensive alterations in proteins regulating organization, cell adhesion, cell-matrix adhesion, and cell movement [10]. Sub-fertile and high-fertile endometrium in pregnancy has different levels of proteins and the mechanisms of pregnancy loss in cows can be associated with impaired conceptus (filamentous vs. tubular) and endometrial (normal vs. subclinical endometritis) communications. 

Interestingly, inclusion of SLC2A1 and SLC27A6 proteins, due to their significant abundances in the GD 16 endometrium and embryo following the transfer of in-vivo GD 7 embryo, in the interactive network analysis revealed links to MX1 and MX2 proteins (Figure S1). It should be noted that MX1 and MX2 expressions caused by embryo-secreted IFN-τ stimulation, were significantly higher in intercaruncular and caruncular endometrium during pre-implantation period (GD 14 to 18) and may be involved in an immune response to protect the conceptus [62]. 

## 5. Conclusions

Candidate genes and proteins elucidated in the current study differed between subclinical endometritis and embryo grade groups. Taken together, embryo-uterine cross talk influences the conceptus survival and programs conceptus development, and effects of dysregulated conceptus-endometrial interaction elicit loss of the conceptus during the implantation period in repeat breeder cattle.

After screening of performance records (*n* = 2389), repeat breeder Holstein cows (Parity 3 and 4), with a history of failing to maintain a pregnancy after the first three inseminations post calving, including current lactation, and with the history of at least one embryo loss between 30 and 60 days (pregnancy was confirmed at pregnancy diagnosis by transrectal ultrasonography at 33 ± 3 days after AI and loss was confirmed at 60 ± 3 days pregnancy diagnosis by transrectal ultrasonography or per-rectal palpation) after any of the first three services during the second or third lactation, were selected (*n* = 681). Endometrial cytology samples from these cows were collected during luteal phase, two weeks before embryo transfer, using a Cytobrush. Cows with SCE (*n* = 180) and no-SCE (*n* = 180) were included in this study. Selected cows served as embryo recipients and randomly received quality Grade 1, 2 and 3 GD7 embryos. Samples from cows were selected, as shown in the figure, for determination of candidate genes’ mRNA and protein expressions. The subclinical endometritis and embryo quality grades class sample cohorts were matched among groups.

## Figures and Tables

**Table 1:** Forward and reverse primer sequence for quantitative real-time polymerase chain reaction amplification of mRNA for bovine conceptus and endometrial samples.

Gene	Primer Sequence (5′–3′)	Product Length	Accession Number
*MUC1*	F: ACCATTGCCTGCAGAAACCT R: CATTGCCCTGGTTGTGTGTC	178	NM_174115.2
*IFNT*	F: CTGAAGGTTCACCCAGACCC R: GAGTCTGTTCATTCGGGCCA	197	NM_001168279.1
*CTSL1*	F: CCTTCACTCCTCCTGACAGC R: TGGTCATGTCACCAAAGGCA	247	NM_001083686.2
*ISG15*	F: TGTCTTTTGAAGGGAGGCCC R: TTATTCACTGCGCTGCATGG	156	NM_174366.1
*RSAD2*	F: GCTGGTACCCATTGCGTTTG R: CTGGCGGGTGAAGTGGTAAT	244	NM_001045941.1
*SLC2A1*	F: CTCATAGCCTGCATCTCGCA R: CCTGTTCCGGAGAGCATTGT	237	NM_174829.3
*CXCL10*	F: CTGCCCACGTGTCGAGATTA R: AAACCGAAGTCCACGGACAA	251	NM_001046551.2
*SLC27A6*	F: TGGAGCACGCAGTGATGTAT R: AAGTCCGGGTTCCCCTTTTT	256	NM_001101169.1
*RXRA*	F: CCTTGACTGCCAGGACTTCTCC R: GGGGGAACTGATGACCGAGA	214	XM_024998424.1
*RXRB*	F: GGAGCCATCTTCGATAGGGT R: GCCTATGGACCTGAGAGCAG	261	NM_001083640.1
*RXRG*	F: GGACACACCCATTGACACCT R: CTTCCAGAAAGATCCCCATCCC	291	NM_001075408.1
*PPARA*	F: GCCCCAGGTGGTGGA R: CCGGCCACAGACTGTTACTT	122	NM_001034036.1
*PPARD*	F: AGTGGCTTCTGTTCACCGAC R: GCTGGAAGGAAGTGAGTGCT	266	NM_001083636.1
*PPARG*	F: CACAGAGATGCCGTTTTGGC R: CAACCATCGGGTCAGCTCTT	173	NM_181024.2
*BRP*	F: CCAGGCTTTAGGCATCACCA	94	NM_001012682
	R: GGCGCCTACTTTGTCTCCTGT		

**Table:** (**a**)

Gene	GD 16 Filamentous Conceptus			Corresponding GD16 Endometrium		
	Embryo QG 1 vs. 3 with SCE	Embryo QG 2 vs. 3 with SCE	Embryo QG 1 vs. 2 with SCE	Embryo QG 1 vs. 3 with SCE	Embryo QG 2 vs. 3 with SCE	Embryo QG 1 vs. 2 with SCE
*PPARA*	0.01	0.01	0.44	0.05	0.05	0.61
*PPARD*	0.01	0.05	0.11	0.01	0.05	0.08
*PPARG*	0.05	0.05	0.46	0.01	0.01	0.10
*RXRA*	0.001	0.01	0.10	0.05	0.05	0.19
*RXRB*	0.05	0.05	0.13	0.01	0.05	0.11
*RXRG*	0.001	0.01	0.10	0.01	0.05	0.17
*IFNT*	0.001	0.05	0.10	0.001	0.01	0.07
*CTSL1*	0.01	0.05	0.15	0.01	0.01	0.05
*ISG15*	0.0001	0.01	0.15	0.0001	0.01	0.37
*RSAD2*	0.05	0.05	0.10	0.05	0.05	0.28
*SLC2A1*	0.0001	0.001	0.05	0.01	0.05	0.10
*CXCL10*	0.0001	0.001	0.01	0.001	0.01	0.08
*SLC27A6*	0.0001	0.001	0.05	0.05	0.05	0.05
*MUC1*	0.0001	0.01	0.22	0.001	0.001	0.13

^1^ Filamentous conceptus, ≥25 mm long; ^2^ Repeat breeder cows were with the history of failing to conceive after the first three inseminations post calving and with the history of at least one pregnancy loss between 30 and 60 days after any of the first three services during the second or third lactation; ^3^ Cows with SCE, >6% PMN; ^4^ Embryo QG: Codes 1 to 4; 1, excellent/good; 2, fair; 3, poor; 4, unfertilized/dead/degenerate); *PPAR*—peroxisome proliferator-activated receptor; *RXR*—retinoid X receptor; *IFNT*—Interferon- τ; *CTSL1*—Cathepsin L 1; *ISG15*—interferon-stimulated gene-15; *RSAD2*—Radical S-Adenosyl Methionine Domain Containing 2; *SLC2A1*—Solute Carrier Family 2 Member 1; *CXCL10*—C-X-C Motif Chemokine Ligand 10; *SLC27A6*—Solute Carrier Family 27 Member 6; *MUC1*—Mucin 1.

**Table:** (**b**)

Gene	GD 16 Filamentous Conceptus			Corresponding GD16 Endometrium		
	Embryo QG 1 vs. 3 without SCE	Embryo QG 2 vs. 3 without SCE	Embryo QG 1 vs. 2 without SCE	Embryo QG 1 vs. 3 without SCE	Embryo QG 2 vs. 3 without SCE	Embryo QG 1 vs. 2 without SCE
*PPARA*	0.05	0.05	0.08	0.05	0.05	0.61
*PPARD*	0.05	0.05	0.34	0.05	0.05	0.43
*PPARG*	0.05	0.05	0.22	0.05	0.01	0.16
*RXRA*	0.01	0.05	0.46	0.01	0.05	0.10
*RXRB*	0.05	0.05	0.24	0.05	0.05	0.58
*RXRG*	0.01	0.05	0.19	0.05	0.05	0.37
*IFNT*	0.01	0.01	0.10	0.01	0.01	0.07
*CTSL1*	0.01	0.05	0.15	0.05	0.01	0.08
*ISG15*	0.001	0.01	0.15	0.001	0.01	0.07
*RSAD2*	0.05	0.05	0.10	0.001	0.05	0.28
*SLC2A1*	0.0001	0.05	0.10	0.05	0.05	0.10
*CXCL10*	0.001	0.01	0.15	0.01	0.01	0.15
*SLC27A6*	0.0001	0.01	0.10	0.05	0.05	0.19
*MUC1*	0.01	0.05	0.22	0.05	0.01	0.13

^1^ Filamentous conceptus, ≥25 mm long; ^2^ Repeat breeder cows were with the history of failing to conceive after the first three inseminations post calving and with the history of at least one pregnancy loss between 30 and 60 days after any of the first three services during the second or third lactation; ^3^ cows without SCE, ≤6% PMN; ^4^ Embryo QG: Codes 1 to 4; 1, excellent/good; 2, fair; 3, poor; 4, unfertilized/dead/degenerate. *PPAR*—peroxisome proliferator-activated receptor; *RXR*—retinoid X receptors; *IFNT*—interferon-τ; *CTSL1*—Cathepsin L 1; *ISG15*—interferon-stimulated gene-15; *RSAD2*—Radical S-Adenosyl Methionine Domain Containing 2; *SLC2A1*—Solute Carrier Family 2 Member 1; *CXCL10*—C-X-C Motif Chemokine Ligand 10; *SLC27A6*—Solute Carrier Family 27 Member 6; *MUC1*—Mucin 1.

**Table:** (**c**)

Gene	GD 16 Tubular Conceptus			Corresponding GD16 Endometrium		
	Embryo QG 1 vs. 3 with SCE	Embryo QG 2 vs. 3 with SCE	Embryo QG 1 vs. 2 with SCE	Embryo QG 1 vs. 3 with SCE	Embryo QG 2 vs. 3 with SCE	Embryo QG 1 vs. 2 with SCE
*PPARA*	0.05	0.05	0.71	0.001	0.01	0.43
*PPARD*	0.001	0.05	0.09	0.001	0.05	0.06
*PPARG*	0.001	0.01	0.06	0.001	0.01	0.08
*RXRA*	0.05	0.01	0.31	0.001	0.05	0.22
*RXRB*	0.001	0.05	0.06	0.001	0.05	0.11
*RXRG*	0.001	0.01	0.10	0.05	0.01	0.17
*IFNT*	0.001	0.01	0.10	0.001	0.01	0.26
*CTSL1*	0.01	0.05	0.15	0.001	0.01	0.07
*ISG15*	0.0001	0.001	0.06	0.0001	0.01	0.34
*RSAD2*	0.05	0.05	0.28	0.05	0.05	0.22
*SLC2A1*	0.05	0.01	0.34	0.01	0.01	0.10
*CXCL10*	0.001	0.001	0.07	0.001	0.01	0.06
*SLC27A6*	0.05	0.001	0.09	0.05	0.05	0.06
*MUC1*	0.01	0.01	0.22	0.001	0.01	0.16

^1^ Tubular conceptus, 10 to 20 mm long; ^2^ Repeat breeder cows were with the history of failing to conceive after the first three inseminations post calving and with the history of at least one pregnancy loss between 30 and 60 days after any of the first three services during the second or third lactation; ^3^ Cows with SCE, >6% PMN. ^4^ Embryo QG: Codes 1 to 4; 1, excellent/good; 2, fair; 3, poor; 4, unfertilized/dead/degenerate). *PPAR*—peroxisome proliferator-activated receptor; *RXR*—retinoid X receptor; *IFNT*—Interferon-τ; *CTSL1*—Cathepsin L 1; *ISG15*—interferon interferon-stimulated gene-15; *RSAD2*—Radical S-Adenosyl Methionine Domain Containing 2; *SLC2A1*—Solute Carrier Family 2 Member 1; *CXCL10*—C-X-C Motif Chemokine Ligand 10; *SLC27A6*—Solute Carrier Family 27 Member 6; *MUC1*—Mucin 1.

**Table:** (**d**)

Gene	GD 16 Tubular Conceptus			Corresponding GD16 Endometrium		
	Embryo QG 1 vs. 3 without SCE	Embryo QG 2 vs. 3 without SCE	Embryo QG 1 vs. 2 without SCE	Embryo QG 1 vs. 3 without SCE	Embryo QG 2 vs. 3 without SCE	Embryo QG 1 vs. 2 without SCE
*PPARA*	0.001	0.001	0.07	0.01	0.05	0.11
*PPARD*	0.05	0.05	0.31	0.01	0.05	0.07
*PPARG*	0.001	0.01	0.06	0.01	0.01	0.09
*RXRA*	0.01	0.05	0.06	0.01	0.05	0.05
*RXRB*	0.01	0.05	0.13	0.01	0.05	0.58
*RXRG*	0.01	0.01	0.08	0.05	0.05	0.37
*IFNT*	0.0001	0.01	0.06	0.001	0.01	0.07
*CTSL1*	0.001	0.001	0.07	0.001	0.001	0.08
*ISG15*	0.0001	0.01	0.09	0.0001	0.05	0.06
*RSAD2*	0.001	0.01	0.06	0.05	0.05	0.08
*SLC2A1*	0.0001	0.001	0.08	0.01	0.05	0.06
*CXCL10*	0.0001	0.01	0.06	0.01	0.01	0.07
*SLC27A6*	0.0001	0.0001	0.06	0.01	0.05	0.09
*MUC1*	0.0001	0.001	0.12	0.01	0.01	0.13

^1^ Tubular conceptus, 10 to 20 mm long; ^2^ Repeat breeder cows were with the history of failing to conceive after the first three inseminations post calving and with the history of at least one pregnancy loss between 30 and 60 days after any of the first three services during the second or third lactation; ^3^ cows without SCE, ≤6% PMN; ^4^ embryo QG: Codes 1 to 4; 1, excellent/good; 2, fair; 3, poor; 4, unfertilized/dead/degenerate); *PPAR*—peroxisome proliferator-activated receptor; *RXR*—retinoid X receptor; *IFNT*—interferon-τ; *CTSL1*—Cathepsin L 1; *ISG15*—interferon-stimulated gene-15; *RSAD2*—Radical S-Adenosyl Methionine Domain Containing 2; *SLC2A1*—Solute Carrier Family 2 Member 1; *CXCL10*—C-X-C Motif Chemokine Ligand 10; *SLC27A6*—Solute Carrier Family 27 Member 6; *MUC1*—Mucin 1.

**Table 3:** *p*-values of pairwise comparisons of protein expressions in gestational day (GD) 16 conceptus ^1^ and corresponding endometrium in repeat breeder cows ^2^ following transfer of different GD 7 embryo quality grades (QG) ^3^.

Gene	GD 16 Conceptus			Corresponding GD 16 Endometrium		
	Embryo QG1 vs. 3	Embryo QG 2 vs. 3	Embryo QG 1 vs. 2	Embryo QG 1 vs. 3	Embryo QG 2 vs. 3	Embryo QG 1 vs. 2
PPARG	0.01	0.05	0.25	0.01	0.01	0.16
RXRG	0.01	0.01	0.34	0.01	0.05	0.37
IFNT	0.01	0.05	0.16	0.01	0.01	0.22
ISG15	0.01	0.05	0.15	0.01	0.001	0.10
SLC2A1	0.01	0.01	0.19	0.05	0.05	0.10
CXCL10	0.01	0.01	0.10	0.01	0.01	0.11
SLC27A6	0.001	0.01	0.08	0.001	0.05	0.10
MUC1	0.01	0.01	0.13	0.01	0.01	0.10

^1^ Filamentous conceptus, ≥25 mm long; Tubular conceptus, 10 to 20 mm long; ^2^ Repeat breeder cows were with the history of failing to conceive after the first three inseminations post calving and with the history of at least one pregnancy loss between 30 and 60 days after any of the first three services during the second or third lactation; ^3^ Embryo QG: Codes 1 to 4; 1, excellent/good; 2, fair; 3, poor; 4, unfertilized/dead/degenerate; PPAR—peroxisome proliferator-activated receptor; RXR—retinoid X receptors; IFNT—interferon- τ; ISG15—interferon-stimulated gene-15; SLC2A1—Solute Carrier Family 2 Member 1; CXCL10—C-X-C Motif Chemokine Ligand 10; SLC27A6—Solute Carrier Family 27 Member 6; MUC1—Mucin 1.

## Data Availability

Data are presented in the main-text and supplementary materials.

## Supplementary Materials

The following are available online at https://www.mdpi.com/article/10.3390/ani11041092/s1; Table S1. Primary, peptide or proteins and secondary antibodies used for determination of proteins. Table S2. Immunoblot lane for quantitative analysis of protein expressions of candidate genes. Figure S1. Selection of embryo and corresponding endometrium for mRNA and protein determination of candidate genes. Figure S2. Interactive pathway of proteins on Day 16 of bovine pregnancy.

## References

[1] Sánchez J.M., Simintiras C.A., Lonergan P. (2019). Aspects of embryo-maternal communication in establishment of pregnancy in cattle. Anim. Reprod..

[2] Diskin M.G., Murphy J.J., Sreenan J.M. (2006). Embryo survival in dairy cows managed under pastoral conditions. Anim. Reprod. Sci..

[3] Diskin M.G., Morris D.G. (2008). Embryonic and early foetal losses in cattle and other ruminants. Reprod. Domest. Anim..

[4] Wiltbank M.C., Baez G.M., Garcia-Guerra A., Toledo M.Z., Monteiro P.L., Melo L.F., Ochola J.C., Santos J.E., Sartori R. (2016). Pivotal periods for pregnancy loss during the first trimester of gestation in lactating dairy cows. Theriogenology.

[5] Diskin M.G., Waters S.M., Parr M.H., Kenny D.A. (2016). Pregnancy losses in cattle: Potential for improvement. Reprod. Fertil. Dev..

[6] Ryan D.P., Prichard J.F., Kopel E., Godke R.A. (1993). Comparing early embryo mortality in dairy cows during hot and cool seasons of the year. Theriogenology.

[7] Sartori R., Sartori-Bergfelt R., Mertens S.A., Guenther J.N., Parish J.J., Wiltbank M.C. (2002). Fertilization and early embryonic development in heifers and lactating cows in summer and lactating and dry cows in winter. J. Dairy Sci..

[8] Wiebold J.L. (1998). Embryonic mortality and the uterine environment in first service lactating dairy cows. J. Reprod. Fertil..

[9] Barnwell C.V., Farin P.W., Ashwell C.M., Farmer W.T., Galphin S.P., Farin C.E. (2016). Differences in mRNA populations of short and long bovine conceptuses on day 15 of gestation. Mol. Reprod. Dev..

[10] Moraes J.G.N., Behura S.K., Geary T.W., Hansen P.J., Neibergs H.L., Spencer T.E. (2018). Uterine influences on conceptus development in fertility-classified animals. Proc. Natl. Acad. Sci. USA.

[11] Fléchon J.E., Flechon B., Degrouard J., Guillomot M. (2007). Cellular features of the extra- embryonic endoderm during elongation in the ovine conceptus. Genesis.

[12] Hoelker M., Held E., Salilew-Wondim D., Schellander K., Tesfaye D. (2013). Molecular signatures of bovine embryo developmental competence. Reprod. Fertil. Dev..

[13] Miravet-Valenciano J.A., Rincon-Bertolin A., Vilella F., Simon C. (2015). Understanding and improving endometrial receptivity. Curr. Opin. Obstet. Gynecol..

[14] Spencer T.E., Forde N., Lonergan P. (2016). Insights into conceptus elongation and establishment of pregnancy in ruminants. Reprod. Fertil. Dev..

[15] Forde N., Lonergan P. (2012). Transcriptomic analysis of the bovine endometrium: What is required to establish uterine receptivity to implantation in cattle?. J. Reprod. Dev..

[16] Forde N., Spencer T.E., Bazer F.W., Song G., Roche J.F., Lonergan P. (2010). Effect of pregnancy and progesterone concentration on expression of genes encoding for transporters or secreted proteins in the bovine endometrium. Physiol. Genom..

[17] Forde N., Carter F., Spencer T.E., Bazer F.W., Sandra O., Mansouri-Attia N., Okumu L.A., McGettigan P.A., Mehta J.P., McBride R. (2011). Conceptus-induced changes in the endometrial transcriptome: How soon does the cow know she is pregnant?. Biol. Reprod..

[18] Farin C.E., Imakawa K., Hansen T.R., McDonnell J.J., Murphy C.N., Farin P.W., Roberts R.M. (1990). Expression of trophoblastic interferon genes in sheep and cattle. Biol. Reprod..

[19] Lonergan P. (2007). State-of-the-art embryo technologies in cattle. Soc. Reprod. Fertil. Suppl..

[20] Kasimanickam R., Kasimanickam V., Grende K. (2020). Endometrial expression of various genes (ISGs, PPARs, RXRs and MUC1) on day 16 post-ovulation in repeat breeder cows, with or without subclinical endometritis. Theriogenology.

[21] Kasimanickam R., Kasimanickam V. (2020). IFNT, ISGs, PPARs, RXRs and MUC1 in day 16 embryo and corresponding endometrium of repeat-breeder cows, with or without subclinical endometritis. Theriogenology.

[22] Kasimanickam R., Duffield T.F., Foster R.A., Gartley C.J., Leslie K.E., Walton J.S., Johnson W.H. (2005). A comparison of the cytobrush and uterine lavage techniques to evaluate endometrial cytology in clinically normal postpartum dairy cows. Can. Vet. J..

[23] Dubuc J., Duffield T.F., Leslie K.E., Walton J.S., LeBlanc S.J. (2010). Definitions and diagnosis of postpartum endometritis in dairy cows. J. Dairy Sci..

[24] Jones G.M., Wildman E.E., Troutt H.F., Lesch T.N., Wagner P.E., Boman R.L., Lanning N.M. (1982). Metabolic profiles in Virginia dairy herds of different milk yields. J. Dairy Sci..

[25] Edmonson A.J., Lean I.J., Weaver L.D., Farver T., Webster G. (1989). A body condition scoring chart for Holstein dairy cows. J. Dairy Sci..

[26] Bo G.A., Mapletoft R.J. (2012). Evaluation and classification of bovine embryos. Anim. Reprod..

[27] Peralta O.A., Huckle W.R., Eyestone W.H. (2012). Developmental expression of the cellular prion protein (PrP(C)) in bovine embryos. Mol. Reprod. Dev..

[28] Betteridge K.J., Eaglesome M.D., Randall G.C., Mitchell D. (1980). Collection, description and transfer of embryos from cattle 10–16 days after oestrus. J. Reprod. Fertil..

[29] Ribeiro E.S., Monteiro A.P.A., Bisinotto R.S., Lima F.S., Greco L.F., Ealy A.D., Thatcher W.W., Santos J.E.P. (2016). Conceptus development and transcriptome at preimplantation stages in lactating dairy cows of distinct genetic groups and estrous cyclic statuses. J. Dairy Sci..

[30] Kasimanickam V., Kasimanickam R. (2014). Exogenous retinoic acid and cytochrome P450 26B1 inhibitor modulate meiosis-associated genes expression in canine testis, an in vitro model. Reprod. Domest. Anim..

[31] Szklarczyk D., Morris J.H., Cook H., Kuhn M., Wyder S., Simonovic M., Santos A., Doncheva N.T., Roth A., Bork P. (2016). The STRING database in 2017: Quality-controlled protein-protein association networks, made broadly accessible. Nucleic Acids Res..

[32] Mi H., Muruganujan A., Thomas P.D. (2013). PANTHER in 2013: Modeling the evolution of gene function, and other gene attributes, in the context of phylogenetic trees. Nucleic Acids Res..

[33] Thomas P.D., Campbell M.J., Kejariwal A., Mi H., Karlak B., Daverman R., Diemer K., Muruganujan A., Narechania A. (2003). PANTHER: A library of protein families and subfamilies indexed by function. Genome Res..

[34] Salilew-Wondim D., Holker M., Rings F., Ghanem N., Ulas-Cinar M., Peippo J., Tholen E., Looft C., Schellander K., Tesfaye D. (2010). Bovine pretransfer endometrium and embryo transcriptome fingerprints as predictors of pregnancy success after embryo transfer. Physiol. Genom..

[35] Hill J., Gilbert R. (2008). Reduced quality of bovine embryos cultured in media conditioned by exposure to an inflamed endometrium. Aust. Vet. J..

[36] Betsha S., Hoelker M., Salilew-Wondim D., Held E., Rings F., Grosse-Brinkhause C., Cinar M.U., Havlicek V., Besenfelder U., Tholen E. (2013). Transcriptome profile of bovine elongated conceptus obtained from SCNT and IVP pregnancies. Mol. Reprod. Dev..

[37] O’Hara L., Forde N., Kelly A.K., Lonergan P. (2014). Effect of bovine blastocyst size at embryo transfer on day 7 on conceptus length on day 14: Can supplementary progesterone rescue small embryos?. Theriogenology.

[38] Sponchiado M., Gomes N.S., Fontes P.K., Martins T., Del Collado M., Pastore A.A., Pugliesi G., Nogueira M.F.G., Binelli M. (2017). Pre-hatching embryo-dependent and -independent programming of endometrial function in cattle. PLoS ONE.

[39] Passaro C., Forde N., Spencer T.E., Lonergan P. (2016). Proteomic analysis of uterine luminal fluid on day 7 of pregnancy in cattle. Reprod. Fert. Dev..

[40] Gray C.A., Abbey C.A., Beremand P.D., Choi Y., Farmer J.L., Adelson D.L., Thomas T.L., Bazer F.W., Spencer T.E. (2006). Identification of endometrial genes regulated by early pregnancy, progesterone, and interferon tau in the ovine uterus. Biol. Reprod..

[41] Klein C., Bauersachs S., Ulbrich S.E., Einspanier R., Meyer H.H.D., Schmidt S.E.M., Reichenbach H.D., Vermehren M., Sinowatz F., Blum H. (2006). Monozygotic twin model reveals novel embryo-induced transcriptome changes of bovine endometrium in the pre-attachment period. Biol. Reprod..

[42] Sofia H.J., Chen G., Hetzler B.G., Reyes-Spindola J.F., Miller N.E. (2001). Radical SAM, a novel protein superfamily linking unresolved steps in familiar biosynthetic pathways with radical mechanisms: Functional characterization using new analysis and information visualization methods. Nucleic Acids Res..

[43] Song G., Bazer F.W., Spencer T.E. (2007). Pregnancy and interferon tau regulate RSAD2 and IFIH1 expression in the ovine uterus. Reproduction.

[44] Berger J., Moller D.E. (2002). The mechanisms of action of PPARs. Annu. Rev. Med..

[45] Shahbazi M.N. (2020). Mechanisms of human embryo development: From cell fate to tissue shape and back. Development.

[46] Bazer F.W., Wang X., Johnson G.A., Wu G. (2015). Select nutrients and their effects on conceptus development in mammals. Anim. Nutr..

[47] Gao H., Wu G., Spencer T.E., Johnson G.A., Li X., Bazer F.W. (2009). Select nutrients in the ovine uterine lumen. I. Amino acids, glucose, and ions in uterine lumenal flushings of cyclic and pregnant ewes. Biol. Reprod..

[48] Kim J.Y., Burghardt R.C., Wu G., Johnson G.A., Spencer T.E., Bazer F.W. (2011). Select nutrients in the ovine uterine lumen. VII. Effects of arginine, leucine, glutamine, and glucose on trophectoderm cell signaling, proliferation, and migration. Biol. Reprod..

[49] Bazer F.W., Wu G., Spencer T.E., Johnson G.A., Burghardt R.C., Bayless K. (2010). Novel pathways for implantation and establishment and maintenance of pregnancy in mammals. Mol. Hum. Reprod..

[50] Ribeiro E.S., Santos J.E.P., Thatcher W.W. (2016). Role of lipids on elongation of the preimplantation conceptus in ruminants. Reproduction.

[51] Ribeiro E.S., Greco L.F., Bisinotto R.S., Lima F.S., Thatcher W.W., Santos J.E. (2016). Biology of preimplantation conceptus at the onset of elongation in dairy cows. Biol. Reprod..

[52] Wieser F., Waite L., Depoix C., Taylor R.N. (2008). PPAR action in human placental development and pregnancy and its complications. PPAR Res..

[53] Cucchi D., Camacho-Muñoz D., Certo M., Pucino V., Nicolaou A., Mauro C. (2019). Fatty acids - from energy substrates to key regulators of cell survival, proliferation and effector function. Cell Stress.

[54] Mattos R., Staples C.R., Thatcher W.W. (2000). Effects of dietary fatty acids on reproduction in ruminants. Rev. Reprod..

[55] Teeli A.S., Sheikh P.A., Patra M.K., Singh D., Kumar B., Kumar H., Singh S.K., Verma M.R., Krishnaswamy N. (2019). Effect of dietary n-3 polyunsaturated rich fish oil supplementation on ovarian function and interferon stimulated genes in the repeat breeding cow. Anim. Reprod. Sci..

[56] Giller K., Drews B., Berard J., Kienberger H., Schmicke M., Frank J., Spanier B., Daniel H., Geisslinger G., Ulbrich S.E. (2018). Bovine embryo elongation is altered due to maternal fatty acid supplementation. Biol. Reprod..

[57] Kasimanickam R., Kasimanickam V., Kumar N., Reisenauer C. (2021). Day 7 embryo quality and suboptimal uterine environment influences morphometry of day 16 embryos in dairy cows. Theriogenology.

[58] Mann G.E., Fray M.D., Lamming G.E. (2006). Effects of time of progesterone supplementation on embryo development and interferon-tau production in the cow. Vet. J..

[59] Brooks K.E., Burns G.W., Spencer T.E. (2015). Peroxisome proliferator activator receptor gamma (PPARG) regulates conceptus elongation in sheep. Biol. Reprod..

[60] Yelich J.V., Pomp D., Geisert R.D. (1997). Detection of transcripts for retinoic acid receptors, retinol-binding protein, and transforming growth factors during rapid trophoblastic elongation in the porcine conceptus. Biol. Reprod..

[61] Bauersachs S., Wolf E. (2012). Transcriptome analyses of bovine, porcine and equine endometrium during the pre-implantation phase. Anim. Reprod. Sci..

[62] Shirozu T., Sasaki K., Kawahara M., Yanagawa Y., Nagano M., Yamauchi N., Takahashi M. (2016). Expression dynamics of bovine MX genes in the endometrium and placenta during early to mid-pregnancy. J. Reprod. Dev..

