# Supplementary material for: mRNA Expressions of Candidate Genes in Gestational Day 16 Conceptus and Corresponding Endometrium in Repeat Breeder Dairy Cows with Suboptimal Uterine Environment Following Transfer of Different Quality Day 7 Embryos
Source: Animals (Basel). 2021 Apr 11;11(4):1092. doi: 10.3390/ani11041092 (PMC8070175; doi:10.3390/ani11041092)
Supplement: Supplementary file 1 [file animals-11-01092-s001.pdf]

Table S1. Primary, peptide or proteins and secondary antibodies used for determination of proteins.

Targets	Primary Antibody	Secondary Antibody
IFN-tau	Mouse anti-tau monoclonal (sc-32274)	Goat anti-Mouse IgG H&L-FITC (ab6785)
ISG15	Rabbit anti-ISG15 monoclonal (7H29L24)†	Mouse anti-rabbit IgG-FITC (sc-2359)
CXCL10	Rabbit anti-CXCL10 polyclonal (PB0385B-100)‡	Mouse anti-rabbit IgG-FITC (sc-2359)
PPARG	Rabbit anti-PPARG polyclonal (AV32880)§	Mouse anti-rabbit IgG-FITC (sc-2359)
RXRG	Rabbit anti-RXRG polyclonal (AV 45631)§	Mouse anti-rabbit IgG-FITC (sc-2359)
SLC2A1	Rabbit anti-SLC2A1(GLUT1) polyclonal (PA1-1063)Ø	Mouse anti-rabbit IgG-FITC (sc-2359)
SLC27A6	Rabbit anti-SLC27A6 polyclonal (SAB2102195)§	Mouse anti-rabbit IgG-FITC (sc-2359)
MUC1	Rabbit anti-MUC1 Polyclonal (ABIN2776819)	Mouse anti-rabbit IgG-FITC (sc-2359)
GAPDH	Mouse anti-GAPDH monoclonal (sc-166545)	Goat anti-Mouse IgG H&L-FITC (ab6785)

Ab – Abcam; ABIN—Antibodies-Online Inc.; †Invitrogen; ‡Kingfisher Biotech Inc.; sc – SantaCruz Inc.; §Sigma-Aldrich; ØThermo Fisher Scientific; IFNT – interferon-τ; ISG15—interferon-stimulated gene-15; CXCL10—C-X-C Motif Chemokine Ligand 10; PPAR—peroxisome proliferator-activated receptor; RXRG—retinoid X receptor gamma; SLC2A1—Solute Carrier Family 2 Member 1; SLC27A6 – Solute Carrier Family 27 Member 6; MUC1—Mucin 1; GAPDH—glyceraldehyde 3-phosphate dehydrogenase.

Table S2. Immunoblot lane for quantitative analysis of protein expressions of candidate genes.

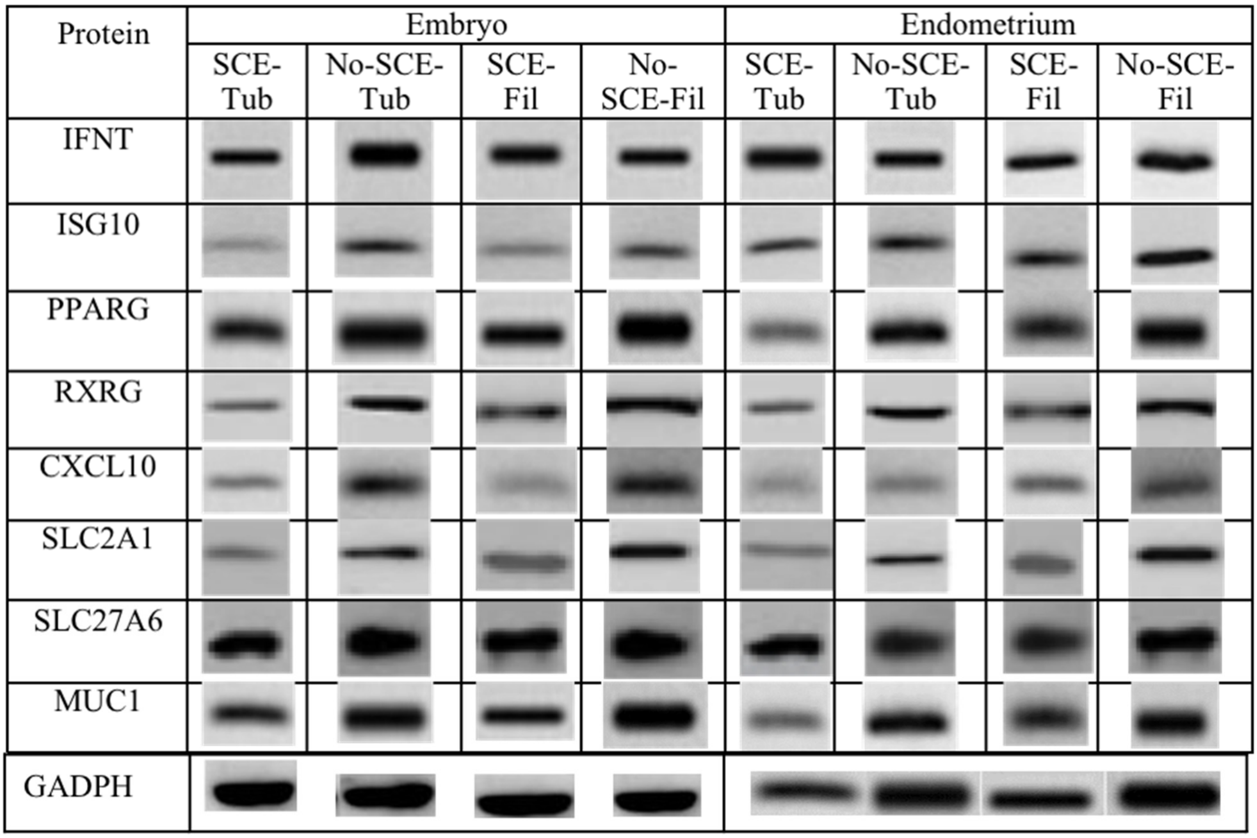

Quantitative protein expression was performed for three separate analysis using the Image J software (National Institutes of Health, Bethesda, MD, USA). The normalized relative protein levels were expressed as arbitrary units (mean ± SE, refer Table 2a–c). SCE, cows with subclinical endometritis, >6% PMN on endometrial cytology; no-SCE, cows without subclinical endometritis, ≤6%; PMN on endometrial cytology; Fil, filamentous conceptus, ≥25 mm long; Tub, tubular conceptus, 10 to 20 mm long.

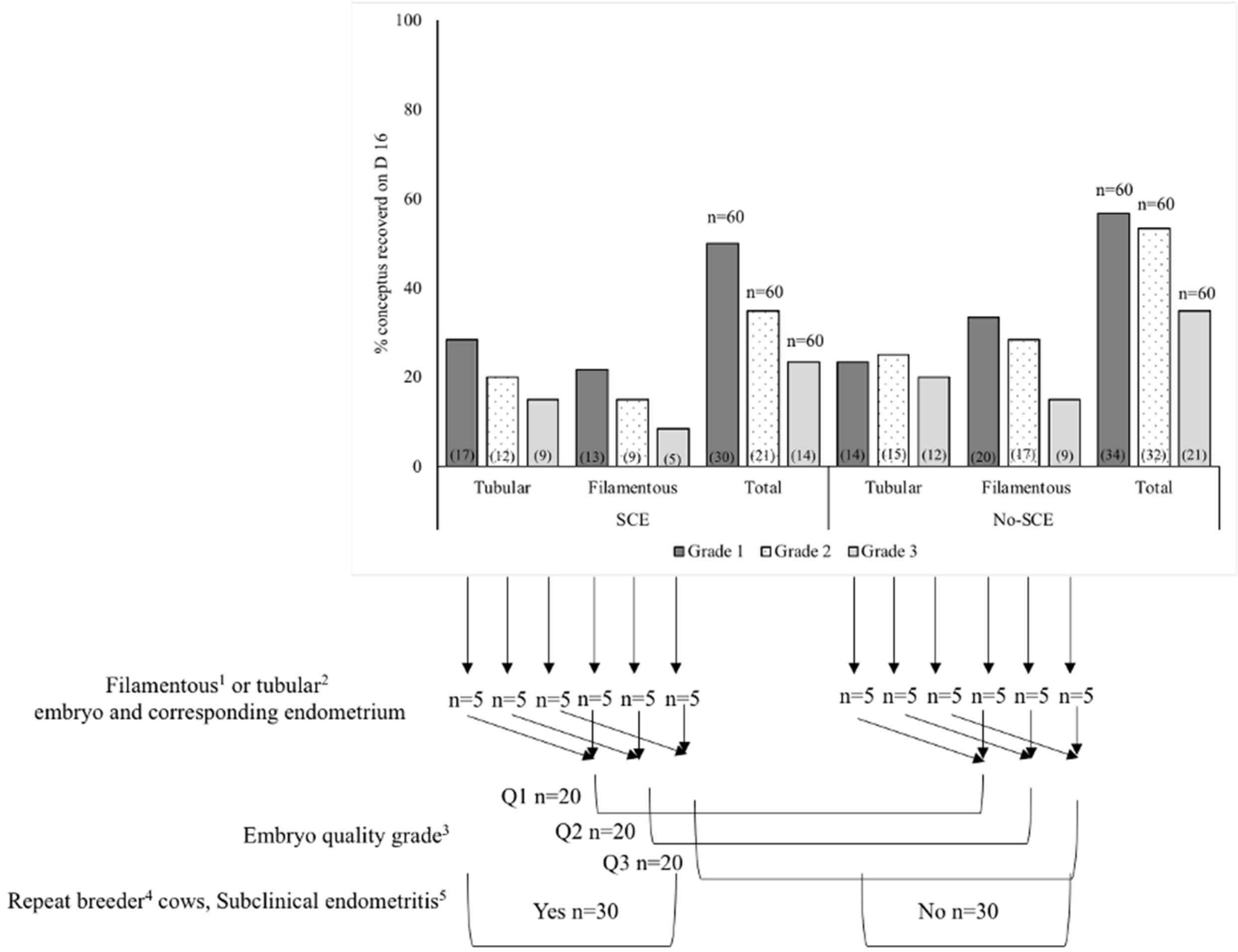

Figure S1. Selection of embryo and corresponding endometrium for mRNA and protein determination of candidate genes. [1] Filamentous conceptus, ≥25 mm long; [2] tubular conceptus, 10 to 20 mm long; [3] embryo grade: Codes 1 to 4; 1, excellent/good; 2, fair; 3, poor; 4, unfertilized/dead/degenerate); [4] repeat breeder cows were with the history of failing to conceive after the first three inseminations post calving and with the history of at least one pregnancy loss between 30 and 60 days after any of the first three s vices during the second or third lactation. [5] Cows with SCE, >6% PMN; cows with no-SCE, ≤6% PMN. Numbers in the parenthesis indicates number of GD 16 conceptus recovered.

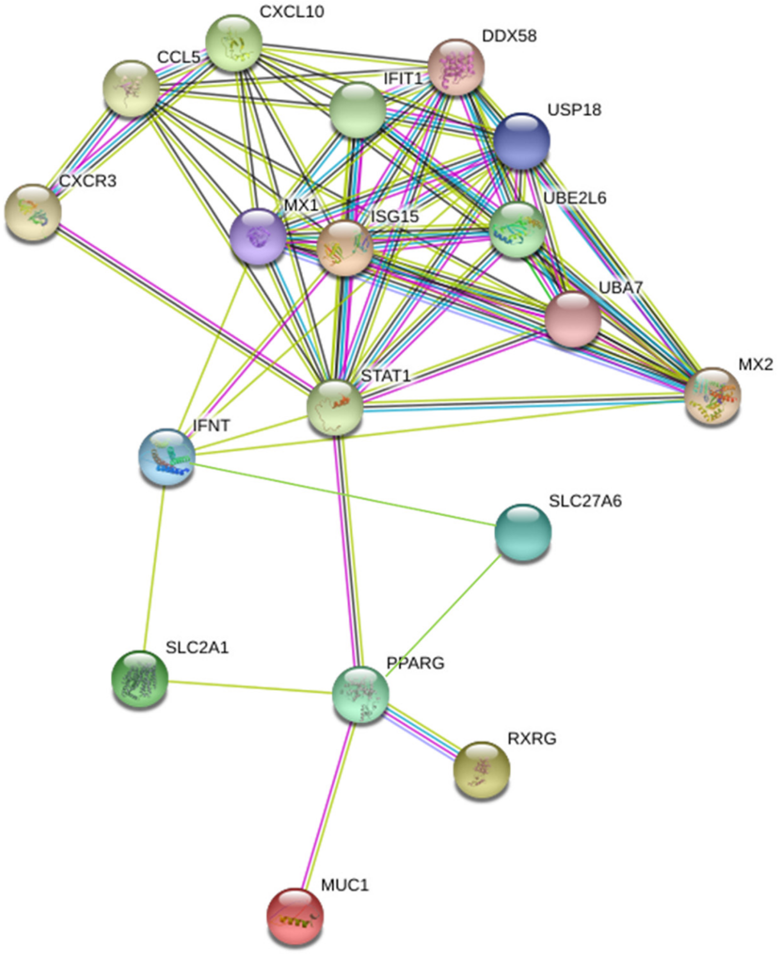

Figure S2. Interactive pathway of proteins on Day 16 of bovine pregnancy.
